# Mechanisms of Dysregulated Antibody Response in Lyme Disease

**DOI:** 10.3389/fcimb.2020.567252

**Published:** 2020-10-07

**Authors:** Timothy J. Sellati, Dana M. Barberio

**Affiliations:** ^1^Global Lyme Alliance, Inc., Stamford, CT, United States; ^2^Edge Bioscience Communications, Sherborn, MA, United States

**Keywords:** Lyme disease, *Borrelia burgdorferi*, antigen-presenting cell, B cell, antibody

## Introduction

Lyme disease (LD), caused by the spirochetal bacterium *Borrelia burgdorferi*, is transmitted by the black-legged tick *Ixodes scapularis* (Hu, 2016). LD is the fastest growing global tick-borne disease and annually affects >300,000 people in the U.S. alone (Steere et al., 2016). The economic impact is a staggering $1.3 billion dollars per year (Adrion et al., 2015). LD can cause long-term, debilitating symptoms, including arthritis, carditis, and neurological complications (Hu, 2016; Steere et al., 2016). A longstanding question is why antibodies produced during primary infection are not able to completely clear spirochetes or confer protective immunity (Barbour et al., 2008). Antibody titers can remain for years in some LD patients while in others, they wane over time or never develop at all (Kalish et al., 2001). Herein, we describe animal studies that reveal mechanisms behind dysregulated development of adaptive immunity and provide insights that may be relevant to human immunity to *B. burgdorferi* infection.

## Role of Lymphocytes and Antibodies in Lyme Disease Pathogenesis

Antibodies produced through B and T cell interactions either within or outside germinal centers are termed T cell-dependent (TD) whereas those produced without the aid of T cells are T cell-independent (TI). One mechanism whereby *B. burgdorferi* attempt to evade adaptive immunity is by continuously changing the sequence of a unique surface protein called variable major protein-like sequence (VlsE) (Norris, 2015). Such sequence variation generates a large repertoire of antigenically-distinct spirochetes that become unrecognizable to the antibodies that are mounted against a previous version of this protein. This may allow *B. burgdorferi* to persist for months or years if not effectively cleared through innate immune response and/or early diagnosis and treatment with antibiotics. Another defensive strategy relies on switching which immunogenic proteins are surface expressed [e.g., Outer Surface Protein A (OspA) and OspC], as spirochetes transit from one environment to another. OspA and OspC are predominately expressed when spirochetes are in the tick vs. the mammalian host, respectively. Notably, whole-proteome microarray analysis revealed that while relatively few *B. burgdorferi* open reading frames (~15%) encode immunogens, those that do elicit the same detectable antibodies in naturally infected humans and wild white-footed mice (*Peromyscus leucopus*), the predominant maintenance reservoir for *B. burgdorferi* (Barbour et al., 2008). Interestingly, a spectrum of disease severity has been observed in different mouse strains, reflecting their unique genetic composition, which controls the magnitude of humoral responses during *B. burgdorferi* infection (Weis et al., 1999).

Despite strong antibody responses in animals experimentally-infected with *B. burgdorferi* and in many LD patients, this does not translate to robust disease-resolving and long-term immunity (Barthold and Bockenstedt, 1993; Aguero-Rosenfeld et al., 1996). In order to explore the mechanisms through which *B. burgdorferi* infection impacts the immune system and gain an understanding of the role of B and T cells in LD pathogenesis, researchers have conducted studies in mice lacking either or both of these lymphocyte populations.

Pathologies associated with *B. burgdorferi* infection of mice often spontaneously resolve, although animals may never completely clear spirochetes. In contrast, after infection with *B. burgdorferi*, severe combined immunodeficient (SCID) and recombination activating gene (RAG)-deficient mice, both of which lack B and T cells, developed severe, persistent arthritis that remained unresolved (Hastey et al., 2012). While B cells regulate disease progression and resolution in wild-type mice (McKisic and Barthold, 2000), adoptive transfer of CD4+ T cells into RAG-deficient mice prior to *B. burgdorferi* infection increased arthritis and carditis severity (unless B cells were co-transferred), and CD8+ T cell transfer increased arthritis severity (McKisic et al., 2000). Conversely, adoptive transfer of serum from immunocompetent *B. burgdorferi*-infected mice into SCID mice ameliorated both arthritis and carditis (Barthold et al., 1997; McKisic and Barthold, 2000). Transfer of immune serum into naive recipient mice either prior to or at the time of inoculation also prevented *B. burgdorferi* infection (Barthold et al., 1997). Immunization of mice with late-stage LD patient sera that demonstrated strong antibody reactivity to several *B. burgdorferi* proteins, including OspA and B, provided partial protection against *B. burgdorferi* challenge (Fikrig et al., 1994). These findings reveal that humoral immune responses generated in experimentally-infected mice and LD patients play an important role in the resolution of some of the most commonly reported clinical manifestations (arthritis and carditis), which are driven principally by activation of inflammatory T cells and release of potent inflammatory mediators.

Researchers found unusually strong and persistent TD and TI IgM antibody production in lymph nodes during early infection and in bone marrow later on in the course of murine infection (Hastey et al., 2012; Richards et al., 2015). IgG-secreting plasma cells, on the other hand, accumulate slowly in the bone marrow. Only about 50% of the IgG response is clearly TD and, coupled with IgM, is thought to contribute to the reduction but not elimination of *B. burgdorferi* from tissues (Hodzic et al., 2003). This TD repertoire of IgG contributes minimally to long-term antibody-mediated immunity, unlike the typical humoral response to bacterial pathogens (Hastey et al., 2012; Tracy and Baumgarth, 2017).

## Mechanism(s) of Immune Dysfunction in Lyme Disease

To dissect the mechanisms behind this dysregulated response, Hastey et al. (2012) elucidated distinct stages of altered immune response using a mouse model of LD. In the first phase of infection, B cells accumulated in lymph nodes and induced antibodies in a TI manner and in the absence of germinal centers. In other infectious diseases, such as mumps and HIV, swollen lymph nodes are a frequent early symptom of infection. Normally, the areas in which T and B cells are found in lymph nodes are well-defined. However, in *B. burgdorferi-*infected mice, this typical architecture was disrupted, with loss of organized B cell follicles and T cell zones (Tunev et al., 2011). Deterioration of B cell follicles, between days 5 and 10 post-infection occurred together with the presence of spirochetes within the lymph nodes (Hastey et al., 2014). In addition, B cells began to accumulate in large numbers, reaching over 70% in some instances and disrupting normal T/B cell ratios (Hastey et al., 2012).

In the second phase, roughly 2–3 weeks later, short-lived germinal centers developed in lymph nodes. These germinal centers gave rise to relatively few antibody-producing plasma cells within bone marrow, leading to a third phase in which plasma cells only slowly accumulated. Lymph node germinal centers disappeared about 1 month after infection, despite the continued presence of bacteria at these sites. Curiously, B cell accumulation occurred after, not before, destructive changes in lymph node morphology. This suggests that the *Borrelia* infection, not B cell accumulation, somehow drives lymphoid tissue atrophy (Hastey et al., 2014).

So, while *B. burgdorferi* infection prompts strong serum antibody levels, and titers increase over the course of infection, the antibody response is ultimately ineffective in completely eradicating spirochetes and/or establishing long-term immunity (Tunev et al., 2011; Hastey et al., 2012; Elsner et al., 2015). *B. burgdorferi* benefits from this maladaptive immune response. This premise is corroborated by a study of antibiotic-treated human LD patients, which included patients who had persistent symptomatology and those who had returned to health within 6 months after diagnosis and treatment (Blum et al., 2018). The researchers focused on plasmablasts, activated B cells that mature into plasma cells within germinal centers. They found that patients who ultimately recovered their health, as compared to those with persistent symptoms, had significantly more plasmablasts during early infection. The researchers determined this by comparing the percentage of plasmablasts as a total of all B cells at the initial clinic visit, during early infection (even before beginning Doxycycline therapy). In addition, patients who ultimately returned to health had significantly higher titers of antibodies to a diverse array of *B. burgdorferi* proteins (Blum et al., 2018). Taken together, this is evidence that *B. burgdorferi* infection redirects the adaptive immune system away from a long-term protective antibody response and toward a less efficacious, rapid and strong, though short-lived antibody response (Richards et al., 2015).

Interestingly, Hastey et al. (2014) also provided evidence that *Borrelia* infection itself may have broader immunosuppressive effects. They tested this by using influenza virus vaccination as a tool to study TD antibody responses. Two groups of mice were influenza-vaccinated, with one group being infected with *B*. *burgdorferi* while the other was not. For the first 3 weeks, both groups of mice produced similar amounts of influenza-specific IgG. However, by 4 weeks, and until the study ended at 26 weeks, the *B. burgdorferi*-infected animals made significantly less influenza-specific IgG than the uninfected mice. By 9 weeks post-infection, there were far fewer influenza-specific antibody-secreting cells in the bone marrow of the *Borrelia*-infected animals compared to uninfected influenza-vaccinated mice (Elsner et al., 2015). This finding engenders an intriguing question about whether LD might negatively impact vaccination efficacy.

## Implications for Diagnostics

There is more to be done in exploring these mechanisms of dysregulated antibody response in LD patients and there are clear implications for development of improved diagnostic tests. Physicians often follow the CDC-recommended two-tiered testing algorithm to detect *B. burgdorferi-*specific antibodies in patients suspected of having LD (Marques, 2015). The first-tier test is an enzyme-linked immunosorbent assay or ELISA, and if results are positive or borderline, a confirmatory second-tier test is done; a Western immunoblot analysis to detect IgM and IgG antibodies that are specific for *B. burgdorferi* proteins. In theory, this test determines if a *B. burgdorferi* infection is active (Marques, 2015). However, this CDC-recommended serodiagnosis may be misleading. In a small study of 79 patients who no longer had symptoms, but had a history of LD with and without arthritis 10–20 years ago, researchers examined antibody titers using the CDC two-tiered test (Kalish et al., 2001). They found that 10 individuals (13%) currently had IgM responses (reflecting initial exposure) to *B. burgdorferi* and 34 (43%) had IgG reactivity (reflecting longer term exposure) to *B. burgdorferi*. Antibody titers were even higher for patients who had LD with arthritis (but were currently asymptomatic), as six of 39 (15%) currently had IgM responses and 24 of 39 (62%) had IgG reactivity. This trend also is seen in infected mice, where IgM antibody levels do not wane but stay relatively high along with the increased IgG response (Hastey et al., 2012).

Theoretically, it would be expected that all recovered patients would lack evidence of IgM and many or all would continue to have circulating IgG. The presence of IgM in 13% of patients would be cause for confusion for physicians as the presence of this class of antibody typically wanes with clearance of the pathogen and recovery from infection. Larger studies need to be done to confirm and to explain the reasons for the continued presence of IgM. Another consideration is that high antibody levels, as discussed, may only offer transient protection, with alterations in germinal center architecture and defective production of long-lived plasma cells and memory cells leading to poor immunoprotection in the long-term (Hastey et al., 2012; Elsner et al., 2015).

Future efforts in Lyme disease diagnosis need to focus on distinguishing between active and inactive infection and improving sensitivity in detecting early disease while maintaining high specificity. Diagnosis would be greatly enhanced with the development and broad adoption of point-of-care testing, and simplified diagnosis. Addition of antigen targets expressed very early in LD (e.g., VlsE1 and pepC10) to current antibody-based diagnostic testing procedures have enhanced performance of the diagnostic assays (Porwancher et al., 2011; Marques, 2015). Additionally, direct detection of *B. burgdorferi* antigens or nucleic acid rather than antibody testing may eventually be possible with the development of advanced technologies (Branda et al., 2018). Not only might direct detection of spirochetal components be indicative of active infection, but the presence of nucleic acids and certain antigens coincides with the earliest stage of infection, when *B. burgdorferi-*specific antibodies have yet to be produced. Examples include *B. burgdorferi* DNA detected using PCR (Mosel et al., 2019) and antigens such as OspC (Ohnishi et al., 2001) or peptidoglycan (Jutras et al., 2019). OspC is expressed on spirochetes as they transit from tick to mammalian host (Ohnishi et al., 2001) while peptidoglycan has been shown to persist in patients long after antibiotic treatment has ceased and patients are theoretically cured of active infection (Jutras et al., 2019). The latter observation strongly supports the notion of persistence of *B. burgdorferi* after antibiotic treatment as peptidoglycan is only produced by metabolically-active spirochetes.

## Discussion

There has been significant progress in deciphering the mechanistic foundation of *B. burgdorferi's* impact on the adaptive immune response, specifically the B cell response and antibody production. While *B. burgdorferi* initially elicits a strong immune response, the end result is a failure by the immune system to clear the infection. This could set the stage for *B. burgdorferi's* persistence (Tracy and Baumgarth, 2017), which may underlie chronic symptoms such as arthritis, carditis, and skin and neurological complications. The many animal studies conducted to date reveal that *B. burgdorferi* relies upon multiple strategies to evade and disrupt the normal functioning of the immune system. The end results are inhibition of effective B cell responses, disruption of the formation of stable germinal centers, and dampening the production of optimally protective antibodies and establishment of long-term memory cell populations. Importantly, many of these same evasion strategies appear to be employed by *B. burgdorferi* in LD patients, particularly those suffering from persistent or chronic disease. These intriguing observations provide an excellent foundation and springboard for further animal and human studies, with the goal of increased understanding of LD pathogenesis, better diagnostics, and ultimately novel and more effective therapeutic options for long-suffering patients.

## Author Contributions

DB and TS contributed to the writing of this manuscript. All authors contributed to the article and approved the submitted version.

## Conflict of Interest

DB is the owner of Edge Bioscience Communications, a freelance, contract scientific/medical writing and consulting company. The remaining author declares that the research was conducted in the absence of any commercial or financial relationships that could be construed as a potential conflict of interest.
